# American Journal of the Medical Sciences

**Published:** 1840-12

**Authors:** 


### REVIEWS.

---

Art. V.—*American Journal of the Medical Sciences. No. LI. Article XII, a Review of Gross's "Elements of Pathological Anatomy,"* under the signature of T. S.

We are told by the Dean of St. Patrick's, that

> "*No goose was e'er so gray but, soon or late*
> "*She found some honest gander, for her mate.*"

And at the time when the sentiment was thus expressed, it was probably true. "*At tempora mutantur, et nos mutamur cum illis.*" Times and their concomitants have undergone such changes, that the truth of the distich, if not quite extinguished, has grown rusty and questionable. Of this, were the honest Dean living at present, we think we might so far convince him, as to induce him to alter or disavow the couplet, or expunge it from his works. And the argument employed should be ocular demonstration.

We have now before us, in the form of an issue from a Philadelphia press, *a thing* (whether "*gray*" or *grisley*, let others decide) of *deliberately meditated calumny and mischief*, not only heretofore *umated* in its kind, but, as we hope and believe, destined to prove *unmatable* hereafter. We allude to the *pasquinade* on Gross's "Elements" (miscalled a "review" of them) referred to in the heading of this article.

Considered in the aggregate, that paper is one of the most repulsive and reprehensible productions of the sort we have ever examined. Some book-knowledge, shrewdness, and independence of mind excepted (yet, instead of "independence," we should have said *effrontery*) it presents a mass of offensive and condemnable matter, destitute of a single redeeming quality. Or, if there belong to it another element approaching an exception to this, it is its style of composition. And though that possesses nothing even bordering on excellence, but is throughout a thing of the meagerest mediocrity, it is so far free from gross and striking faults, as to be entitled to pass without censure, protected from the critic's scourge by its insignificance.

The spirit of ill-natured cavil, censoriousness, and acrimonious but edgeless sarcasm, which breathes through the article, from beginning to end, is, in the utmost degree, *condemnable*, not to apply to it a harsher term; and, to all liberal and well-disposed readers, that spirit must necessarily render it extremely disgusting. To discourteous and embittered calumniators only can it be acceptable.

True; the paper neither pours forth a constant stream, nor breaks out in fitful explosions, of consuming ire, as if it would reduce to ashes, and scatter to the wind whatever may oppose it, or dissent in any way from its oracular dogmas. But, in its essence, it is intensely rancorous and malignant, and would, were its energy equal to its fellness, wither like the sirocco.

If the article be not instinct with a spirit thus deeply malevolent and destructive, then are its internal qualities at utter variance with its external aspect. For such is the import of its ill-boding physiognomy. But we must speak of it in simpler and less figurative terms.

Previously however to our commencement of the exposition here contemplated, we shall give a succint representation of the light in which the task that lies before us is to be viewed; that it may be the more fully and clearly understood, and the more readily and justy appreciated by our readers.

It is our wish, then, to have it known, and borne steadily in mind, that the animadversions about to be made on the production we are to examine, will have nothing in them of a private, or personal, or party description—nothing, we mean, of either vindication or censure *exclusively individual.* To whatever extent such *seeming* sorts of matter may be occasionally introduced, they will be *only* seeming, and not *real.* In proof of this, they will be ultimately made subservient to the support and promotion of a *general* purpose. And that purpose is,

THE DEFENCE OF WESTERN MEDICINE AGAINST THE ASSAULTS AND ASPERSIONS OF A FEW EASTERN PENS.

We say "a few" of those "pens;" for, to the honor of our eastern brethren *generally*, and in strict justice to them, we acknowledge that they are *but few.* We even believe them to be nothing more than the wanton instruments, not of any thing sufficiently respectable *in number or standing* to be called *a party;* but of a puny, carping, and self-sufficient *clique*, not in any degree supported by the sympathy, countenance, or co-operation of the high-minded and well-disposed members of the profession in the Atlantic States.

But petty as we believe this evil-eyed bevy to be, it is sufficiently considerable to do mischief, by injustice and traducement, unless it be opportunely chastized, and effectually checked. An issue from it thus injurious becomes the more certain, when, as in the present instance, the assailing body, though feeble in itself, adds to its means of annoyance and aggression, by operating through a Journal of standing and influence, which has sullied its character, by becoming thus the instrument of an ill-intentioned faction. In such a case the authority of the Periodical passes to the credit of the factious collaborators, and in that way magnifies, sanctions and confirms the evil—But, without further preface, we must hasten to our work.

The criticism, by T. S., as there is a strong reason to believe, was the result of a concerted scheme, in the nature of a *conspiracy*. No doubt can be even plausibly entertained, that it was concocted and composed, with a premeditated design to destroy, if possible, the credit and character of Professor Gross's Elements—to degrade them from their present justly elevated standing in public estimation, and thus prevent the work from circulating extensively, and becoming a text-book in Pathological Anatomy—among other reasons, because it is a product of the West, and not of the East. For, in their caviling comments on the writings of *western* physicians, the bevy of *modern Zoiluses*, to which we have referred, seem abundantly imbued with the captious and and persecuting spirit of the *Hebrew inquisitors of old*, when they tauntingly demanded, "Can any thing good come out of Nazereth?" Nor are they backward in applying this arrogant interrogatory sneer, to their own commendation. Had not some feeling to this effect occupied, at the time, the mind of the reviewer, it is hardly possible, that he would have toiled so arduously, and so discreditably equivocated, as he has done, to find in the work, which he professed to be examining, a *few inconsiderable faults*, when he could, with twenty times the ease, have pointed out striking excellencies, *in fifty-fold* the number. For it is a fact as singular as it is suspicious, that, while all other journalists, (and they are *numer-*

*ous*) who have noticed Gross's Elements, have, without an exception, which we now remember, bestowed on the work the highest commendations, for its numerous and varied excellencies, T. S. has commended it, in scarcely a single instance ; and, when he has done so, it has been in a style of the most cold and meager approval—his thoughts and words apparently dragged out of him by a fifty horse-power! Of cordial praise he has given it none.

Nor has the work received honors from *journalists alone.* Far from it. It has been adopted as the *text-book* on Pathological Anatomy, by several of the medical schools of our country. *And Dr. Gerhard has himself* (as we are positively assured) *recommended it to his pupils, in the Blockley Hospital!*—which he certainly would not have done, had his opinion of it been as unfavorable as that of T. S. And, as a farther proof to the same effect, soon after its publication Professor Gross was elected a member of the Philadelphia Pathological Society, of which Dr. Gerhard is President!!

Such are the facts of the case. And for the production of the sweeping and indiscriminate condemnation by the reviewer, a *strong reason* is alleged to have been in action. We refer to such a reason, however, only as matter of apparently well-grounded suspicion and report, without professing to have any intelligence, amounting to positive proof of its existence. But supposing it to have existed, which we have never doubted, whether it was just, magnanimous, or in any way undeserving of deep reprobation, let an enlightened and liberal community judge. And if we mistake not their sentiments in a case of the kind, they will judge severely, and decide promptly. The reason alleged is as follows.

A physician of Philadelphia, of high qualifications, is said to be engaged in the preparation of a work on Pathological

Anatomy, designed also for a *text-book*—at least as a source of elementary instruction for students of medicine, And the super-sensitive reviewer was scandalized even by the *fancy*, and quite indignant at the *fact*, that a Western physician should have the effrontery to publish on the same subject before a Philadelphian!—and thus, perchance, bear away, "at one fell swoop," the palm and the profits!

Hence the probable ground, on which T. S. presumes to rebuke Professor Gross, in a tone and manner, neither decent nor admissible, not to apply to them terms more scornful and reprehensive, for not having devoted *more than four years* to what he calls, with the intent to disparage it, the "COMPILATION" of his work; while he knows, or at least *ought* to know (the fact being true and susceptible of proof) that the production is *not* a compilation; containing as it does, a large amount of matter, *not derived from other writers;* but which the Professor drew from his own resources. And, as respects the time spent by Professor Gross in the preparation of the work, "*four years*," as every body knows, are a protracted period, for an author to devote to the preparation of a couple of volumes, in the United States; especially in these "*heels-over-head*" times, when the brain, the pen, and the press move alike with locomotive speed. On few if any professional work, of the same size, has more time, we think, been bestowed by an American writer. In the present case, moreover, had not the publication of the two volumes, of whose precipitate passage into existence T. S. complains in a style so pert and unbecoming, ante-dated the birth of the Philadelphia production already referred to, and thus threatened to "keep it from the light" and bar against it the doors and avenues of sale and circulation, by itself taking possession of them —had not the appearance of the "Elements" done this, we venture to believe, that their "four years" of gestation would not have been either professionally denounced, or even spoken of unkindly. And we further believe, that, had the volumes in question been a work of *inferior merit*, and therefore not dangerous to the projected Philadelphia publication, they would have experienced from the American Journal much less of censure and intolerance, than has been so liberally awarded them. In minds of a certain caste and caliber, the rancour of the hatred is in direct proportion to the cravenness of the fear, experienced by them toward a powerful and dangerous adversary. It is the brave and magnanimous alone that cherish and express a becoming regard for a distinguished antagonist. The very condemnation therefore so lavishly bestowed on the "Elements of Pathological Anatomy," may be correctly enough received, as a virtual acknowledgment of their peculiar merit. We shall only add, that so gross is the discourtesy of the criticism on those volumes, as to amount, in some instances, to unqualified rudeness, not to strengthen the expression, and call it vulgar insult. If the reviewer is not conscious of the truth of this remark, he is destitute alike of sound judgement, and refined feeling; and if he is thus conscious, and has notwithstanding been guilty of the outrage, he is an unprincipled slanderer. He may take his choice. On one or the other horn of the dilemma he is destined to be empaled.

Admitting the truth of all we have alleged, (and we hold ourselves thoroughly prepared to prove it) what should be said of the "American Journal of the Medical Sciences," which assumes to be the Delphic oracle of medicine for the *United States*, and has invoked aid from western pens in furtherance of its purposes to that effect—what should be said of that Journal, for the promulgation through its pages, under such circumstances, of an article so reprehensible in its spirit and character, and, in its bearing, so unjust and unfriendly to western medicine? To this question an enlightened and magnanimous public will find no difficulty in rendering the merited reply. And that that reply, when given, will be condemnatory of the act, cannot be doubted.

Admit the well known claim of the American Journal, and it is the head of the whole American family of medical Journals, and the *foster parent* of all the other medical productions of the country. What then should be its spirit and deportment toward such productions?—kind, parental, dignified, and encouraging—not marked and degraded by the spiteful taunts, and embittered censure of an exasperated *step-dame.*

In saying that the American Journal has "invoked aid from western pens," it is our wish to be clearly and distinctly understood. And our allusion is particularly to the following occurrence.

In the course of last summer, a distinguished Philadelphia physician, warm in his friendship toward the American Journal, and interested in its standing, passed a few days of sojourn in the city of Louisville. And while here, he made to some of the physicians of the place, a direct proposal to the effect we have stated. He expressed a wish that no medical Journal might be henceforward published west of the mountains; but that western physicians should write only for the "American Journal," in order that the concentrated result of the observation, reflection, experience and every other form of research and information, of the whole American Faculty of medicine,—a Faculty whose resources are furnished by a region more extensive than Europe, and embrace all that concerns the health of sixteen or eighteen millions of people—

in order, we say, that this mighty mass of professional intelligence might be proclaimed to the world, by a SINGLE ORACLE—and that oracle be the AMERICAN JOURNAL!! On such a proposal comment would be lost. The silence of astonishment most fitly corresponds to its *boundless presumptuousness!!*

Notwithstanding this overture, which, though not so intended, was virtually an indignity to the manly ambition and independence of the West, yet in the very first number of that Journal subsequently issued, is contained the article so unjustly and arrogantly censorious and condemnatory of a western publication; though that publication is decidedly one of the most valuable and creditable works in medicine, that has ever been written in the United States! Such is the consistency of some of the friends and immediate patrons and collaborators of the American Journal! and such the bearing of that Periodical toward western medicine! And though we do not pronounce it the *official organ* of any of the Philadelphia medical schools; yet if we did so pronounce it, our apprehension of being convicted of error would be but slight. And a majority of the Faculty of the country would back us in opinion.

Before taking leave of the American Journal, we shall advert to a clause or two contained in its " Advertisement for a New Series," published in the August number of it, and also issued in a Circular. From that paper we make the following extracts.

"The great object proposed in the institution of this Journal" (the American) " was to establish a NATIONAL WORK devoted exclusively to the improvement of medical science, and to the elevation of the character and dignity of the profession, to the entire rejection of all local and individual interests and party views." * * * * * " THE OBJECT AIMED AT HAS BEEN ATTIAINED, AND THIS JOURNAL IS REGARDED BY THE GREAT MASS OF THE AMERICAN

MEDICAL PROFESSION AS THEIR REPRESENTATIVE, AND AS SUCH IS RECEIVED AND QUOTED ABROAD." Again;

"In this department" (that of reviews) "entire freedom of criticism is allowed, always, however, marked by *candour*, and in that *courteous tone*, which alone comports with the *true dignity* of science."

These extracts speak *for* themselves and *of* themselves, with a degree of plainness which renders it hardly possible to misunderstand either their meaning or their object. Our comments on them shall therefore be brief, and clothed in language of the same plainness with that which characterizes our text.

Of the proud aim of the American Journal at a NATIONAL standing, we do not complain. On the contrary we deem it both creditable and useful. Without loftiness of aim, loftiness of attainment, or of action, is never achieved. But of the dogmatical and self-sufficient proclamation of the Periodical, that it has actually "attained the GREAT OBJECT of its institution," (a "national" rank) we cannot speak in terms so commendatory.

Admitting the proclamation to be true, it would, we venture to think, have been much more in accordance with delicacy and decorum, to have placed the announcing trumpet in other hands, and allowed it to be filled by other lips, than those of either the Editor, or the Proprietors of the Journal. Nor might it have been amiss in those gentlemen, when they were preparing for their blast, to have called to mind the time-worn adage, whose truth has stood the test of ages, that "*self-praise is no commendation*." But the worst is to come.

We again, in a tone of entire confidence, venture not only to "think," but openly to assert, that the proclamation is *not true*. The "great mass of the American medical profession" *do not* regard the American Journal as their "REPRESENTA-

TIVE." Much more exclusively (or we are misled by what we have seen and heard) does the medical profession of our country regard that Periodical as the *representative of one of the Philadelphia schools of medicine.*

Be these matters however as they may, we feel secure from contradiction in saying, that through the whole of the first extract there breathes a spirit of *self-glorification* and *complacency,* which does not well comport with sentiments of becoming modesty, and enlightened self-respect. And we have sought in vain for evidence of the Journal's redemption of its pledge to " reject all *local* and *individual* interests and party views." On the contrary, the import of the evidence presented, appears to us directly the reverse.

As regards the last extract, it neither calls for, nor deserves from us any reply or notice, other than that which it has already received. In what degree the American Journal has, in its "review department," redeemed its pledge to observe "*candour*" and "*courtesy* of tone," or "true dignity" of manner, toward the works and their authors subjected to its ordeal, our readers may learn from the strictures we have already made, and some which we shall make hereafter, on the review of Gross's "Elements," by T. S. To that source of information therefore they are respectfully referred.

A thought or two more on the NATIONALITY, and the REPRESENTATIVE character of the American Journal, and we shall be done with it under that head.

The collaborators are of course the REPRESENTATIVES (we might say the VERY LIFE-BLOOD) of the Journal; and (the Editor making one of them) they number *forty-three;* not more, we apprehend, than *one* in every *three* of whom writes for the work a single line *per annum.* The number of actual collaborators, then, does not *exceed fifteen*—we doubt much whether it

*reaches* it—and the *regular* contributors even of these (if there be any) are by no means the ablest writers on the list. Such we believe is a correct statement of the case.

Let then the names of these *fifteen real* collaborators be selected, and presented to the whole "American medical profession," accompanied by the question, "Do you choose these men as your PROFESSIONAL REPRESENTATIVES, in all civilized countries, and so acknowledge them to your contemporaries and to posterity?"—let this we say be done, and what will be the issue? The answer is plain. It will be an emotion of surprise, if not of scorn and scoffing, and a negative reply from Maine to Louisiana, and from the eastern to the western extremes of the United States. And not a few of the collaborators themselves will cordially unite in it. We verily believe that the whole of them will thus unite. There is not an individual among them, who would not consider such a proposition preposterous—or something worse.

We wish it to be understood, that, in making these remarks, we mean no disrespect toward the collaborators of the American Journal, *as individuals.* Our animadversions relate only to the fallacy and folly of pronouncing the writings of *any* fifteen, twenty, or even *fifty* physicians, residing in a *small section* of the United States, to be a fair epitome or representation of the professional views of the whole body of the American Faculty.

As regards the assertion, by the Editor and Proprietors, that the American Journal is "received and quoted *abroad*, as the REPRESENTATIVE of American medicine," whether it be true or false, is a matter of indifference to us. Such reception and quotation, supposing them to exist, have no bearing on the facts of the case. They only unite with other circumstances to show, that the people *abroad*, even the most fully informed of them, are too ignorant of the great medical concerns of the United States, to be competent judges in a question of the kind, which involves such a variety of unsettled elements.

To consummate the untruth and absurdity of pronouncing the American Journal the REPRESENTATIVE of American medicine, a remark or two more will be fully sufficient.

But *one* of the collaborators of that work (Professor Mussey of Cincinnati) resides in the Mississippi Valley, which contains perhaps *six millions of inhabitants.* And his residence in the West has been brief, and his sphere of medical practice and observation too limited to be a source of much information, either to himself or to others. Without intending the slightest disrespect toward the Professor, therefore, we assert that he is utterly unfit to represent the condition of western medicine. He would not himself aspire to an enterprise so arduous. Nor do we know that he has written a line for the American Journal, since his migration to the West. We are rather inclined to believe that he has not. But his having done so would have no effect on the nationality of the work.

And it would be easy to show that the case of Professor Smith of Baltimore, who is also a *nominal* collaborator of the American Journal, is still weaker. True; he has lectured on medicine in Lexington, one of the healthiest towns in the Union, during *two winters.* But he has had no opportunity to see even a single case of the summer and autumnal diseases of the West. To represent him therefore as a qualified representative of western medicine, would be as strongly marked by folly as by presumptuousness! Nor would he, we feel condfident, countenance the act.

We shall only add, that the entire list of the American

Journal's collaborators does not contain the name of a single individual, who, *from his own resources*, is prepared to write a *respectable essay* on the diseases of the Mississippi Valley. Were any one of the collaborators to attempt such a production, he could not possibly succeed in it, without deriving the matter of it from western physicians.

If the foregoing representations be incorrect, let their incorrectness be established, and we shall instantly retract them, on account of their fallacy. And if they be true, we leave it to the decision of an enlightened public, whether the American Journal is not bound, for a similar reason, to retract its claim to the high standing of a NATIONAL REPRESNTATIVE of American medicine, either at *home or abroad?*

That we may not be suspected of exaggerating the odiousness of the spirit of the article before us, we shall extract from it a few of its offensive passages. Nor shall we trouble ourselves by searching for the *most* offensive, as it would not be easy to make the selection—so nearly do many of them come to an equality in demerit.

That the entire force of our statements and comments may be the better understood, and the more correctly appreciated, we shall first lay before our readers the observations of Professor Gross, and follow them immediately by the exceptionable strictures and carpings of the reviewer.

Before commencing this process, however, we ask the favour of our readers to bear in mind what we have already said—that, as far as our knowledge on the subject extends, every Journal, the American excepted, that has noticed Professor Gross's "Elements," has done so most favourably—several of them in terms of superlative commendation. This contrast renders the entire case of the review in the American Journal the more singular and unaccountable, and might justify us in exclaiming with the poet,

"There's something rotten in in the State of Denmark,
"Which needs much reparation!"

A mere difference between men in intellect, attainment, and taste, could never produce a difference so wide and radical in unimpassioned judgment. As soon shall the difference of a few degress in latitude and longitude, produce antipodism in situation. But one cause can account for such a glaring contrariety of sentiment between writers and judges nearly equal in mind, and enjoying nearly similar opportunities to be informed. And that cause is, *justice, impartiality, and kindness on one side; and injustice, hostility, and prejudice on the other.*

Before commencing the extracts, and our comments on them, we shall observe, once for all, that it is not so much the *substance* of the reviewer's remarks and cavils that we deem so reprehensible. It is the odious spirit with which he has made them. And that can be fully perceived and appreciated, in its deformity and offensiveness, only by a perusal of his entire paper. We quite despair therefore of portraying it to the life. We shall only add in this place, that the reason why the objections and censures of T. S. are not *substantially* condemnatory and severe, is sufficiently obvious. It was no want of *desire* in the gentleman to that effect. It was in part perhaps from a want of *power* in him; but chiefly, because he could not find in the work he was endeavouring to injure, matter sufficiently exceptionable to render them so. In returning from this digression, to the more immediate object of our article, we ask the particular attention of our readers to the proposed extracts from the Professor and his reviewer, which shall be laid before them, and are as follows:

Professor Gross. "Bearing in mind the above definition, it may be assumed as a general proposition, liable to few exceptions, that all organic diseases, whatever be their seat or extent, are the result of inflammatory action, either of an acute or a chronic kind. To many this proposition may be startling; nevertheless, if it be carefully examined, it will be found, I doubt not to be grounded on fact."

Reviewer's remarks. "Why our author should think the above proposition might prove *startling* to many, does not seem very clear. It is certainly not so from its novelty, since even *the merest tyro* in medicine is familiar with it as one of the principal dogmas of Broussais. It is we confess however rather *startling* to hear so exclusive a view reiterated at the present time, as a *positive truth*, when its insufficiency is maintained by most pathologists of the present day."

Here the reviewer, *by way of taunt and affected contempt*, twice *repeats* the words of Professor Gross—an act which is never practised by *gentlemen* toward each other, in such a spirit and temper, except as a measure of actual insult, or, which amounts to the same thing, as a manifestation of scorn or disrespect. We have called the reviewer's contempt exhibited toward Professor Gross "affected," because, toward the author of a work like that which he was struggling to discredit and destroy, he could not *feel* contempt, in the true acceptation of the term. Nothing can awaken that feeling in a man's mind, except what he really considers *beneath* him. And, from the unlucky moment, in which T. S. began to empty his *vial of malice and smothered wrath*, on the fair pages of Professor Gross's "Elements," until the last dripping of its noisome dregs, he felt a mortifying consciousness that their author was perched immeasurably *above* him. In evidence of this, had the Professor been on the spot, the reviewer would not have used, in his presence, the words and manner, which he did in his absence. Yet the manly and honorable rule of reviewing is, for the critic never to say *of* his author, what he would shrink from saying *to* him. But to that rule of magnanimity and fairness, *defamers* are strangers. Like the dastardly assassin, they strike from behind.

The reviewer is moreover no less wanting in justice and candour toward Professor Gross, than he is in courtesy and observance. The Professor has not stated, "*as a positive truth*" his *belief* that "all organic diseases are the result of inflammatory action"—though the covert and disingenuous insinuation of the reviewer is to that effect. He has, in his own words, offered it only "as a general proposition, *liable to few exceptions.*" He has no where pronounced it *free from exceptions.* As T. S. therefore justly insists on accuracy in Professor Gross, he ought to sanction and strengthen his exaction, by exhibiting an example of acccuracy in himself. Before he troubles himself to brush the motes out of his neighbour's eye, let him drag the beam out of his own. But of his blindness and blunders more shall be said hereafter. Again.

Professor Gross. "Most writers seem to me to have attached too much importance to them" (the *phenomena* of inflammation,) "and too little to others; whilst they have entirely overlooked the fact, that they are always greatly modified by the nature of the tissue, in which the malady, of which they are the indices, is located."

Reviewer. "If by *most writers* is meant most of those who have written *since the time of Hippocrates*, the assertion will not be denied; but if, as we naturally conclude, is meant most writers of the present day, it is unquestionably erroneous; for the modification of the phenomena of inflammation, according to the tissue affected, is now so universally admitted and appreciated, as to constitute it one of the most important and prominent features of modern medicine."

To readers of good breeding and discernment, we need not remark, that the two or three first lines of this extract are designed by the reviewer to express one of those conceited, coarse, and impertinent sneers, which are so frequently uttered by ill-bred boys, and rude clowns, when they would fain be thought witty. They amount to an out-break of *positive vulgarity!* The whole extract however would be unworthy of notice, were it not that it is characteristic of the snarling and affectedly jeering spirit, which the reviewer has manifested from first to last, in the composition of his paper—a spirit which, having led him, in violation of the decorum of letters, and the sanctity of truth, to attempt the degradation of the books he had before him, has degraded himself. Nor is this all. T. S. has here been guilty of *equivocation.*

Of "*most*" of the works that "have been written since the time of Hippocrates," we presume to know but little. Yet it is quite possible, and perhaps probable, that we know more than the erudite reviewer. Be this however as it may, we *do* know, and he *ought to* know, enough of medical literature (holding as he does the *high post* of COLLABORATOR to the American Journal, and perhaps other titles to distinction, not yet revealed to us)—thus *bedizened* in his *star* and *garter* of medical glory, he *ought not* to be ignorant of the fact, that, in *nearly* all the books written, until a quite *recent period—the period,* we mean, of the labours of Bichat—no competent notice is taken of the "modification of the phenomena of inflammation according to the tissue affected." At present we recollect no such notice in any work composed anteriorly to that time. The reason is plain. Prior to that date comparatively little was known about the distinctive characteristics of the different tissues—not to say about the *existence* of several of them. The hypercriticism of T. S. is therefore, if not in the manner of an *equivoque,* at least as unsound, as his temper is captious, and his disposition at war with candour and justice.—How again can a more direct and impudent indignity be offered in words, than is contained in the following fragments of a paragraph, which is grossly insulting, from beginning to end!

"Were our author" (Professor Gross) "*better acquainted with the laws of abnormal action* (the italicized words are another *taunting repetition* of the Professor's own language,) and had he studied morbid anatomy *less exclusively* in the *dissecting room*, and been a *patient observer* of the symptoms developed during life, he would hardly, we think, have penned the above paragraph." * * * * "Were these opponents (of Professor Gross) disposed to retort, they might perhaps tell him, that *his mind was not sufficiently philosophical to comprehend fully the terms of the question*, or to make a distinction between a lesion being essentially dependent upon inflammation, and its being at times more rapidly developed or hastened in its course by this cause."

Each of these quoted "fragments" is sufficiently *abominable;* but the first, in particular, carries on its brow a brand of infamy, as deep and indelible as was that on the brow of the FIRST FRATRICIDE. And, if such be the *fruit*, what should be said of the *weed* that produced it! To this question, let others reply. Our concern is with the base and mendacious insinuations of the reviewer, respecting the personal qualifications of the writer he has slandered.

In the face of those insinuations, which, in every element, are irredeemably false; and, in the face of the calumniator, who had the audacity to pen them, we confidently aver, that Professor Gross has studied the subject of his work most faithfully and laboriously, in the sick-room, as well as in the dissecting-room. He has therefore been "a patient observer of the symptoms developed during life," the causes of which he has so successfully sought for, by dissection after death. In proof of this, we could adduce an abundance of conclusive testimony, could we stoop to a grave and formal contest, with T. S.—a writer, whom we can recognize, at present, in no other capacity, than that of a reckless traducer of merit; and who, to make the most and best of him, knows nothing of the medical career of Professor Gross—whether he studied "Pathological Anatomy" in the sick-room or the dissecting-room—in public hospitals or private dwellings. We, however in common with hundreds of others, do know something of the matter, and are prepared to testify, that he has faithfully and accurately studied it in all of them. Yet has T. S., without a shadow of testimony to sustain him, overwhelmed himself in the disgrace of an illiberal and unmanly insinuation to the contrary! Under these circumstances we sincerely trust, and shall even venture to implore those concerned in the matter to that effect, that, before any farther efforts shall be made, formally to erect the American Journal into a NATIONAL ORACLE, duly commissioned to announce to the Old world, and to the *whole* world the condition of the profession of medicine in the New—before the consummation of this national enterprise be farther attempted, we would most earnestly invoke the learned Editor of the American Journal, to strike from his roll the name, and dispense with the farther services of every collaborator, who writes on topics of which he is ignorant, disregards truth, and obeys passion and prejudice instead of reason and judgment; and, more especially, who, in contumacious rejection of these, and all other like considerations, strenuously endeavors to cover with disrepute the *most useful work* on Pathological Anatomy, now extant in the English language—and, as we verily believe, *in any language*. And the work thus slandered being an *American* production, the slanderer is the more deeply unworthy to minister, as a sarcerdotal functionary, at the shrine of the American ORACLE IN MEDICINE.

Once more. "All," says the reviewer, "that is valuable in the work (Gross's Elements) might, with advantage, have been comprehended in *half the space* it now occupies."

This charge can receive but one answer. IT IS UNTRUE. The work does not contain more matter than is requisite to fit it for the end for which it is intended. Nor does it contain any thing irrelevant to that end. Or if it does either the one or the other, or both, T. S. will equally oblige and instruct us, by specifying in what the surplus or the irrelevancy consists. And though we do not pronounce the style of it faultless, nor say that it might not be somewhat condensed and abbreviated, by the labour of pruning; we do say that it is neither unusually verbose, nor exceptionably diffuse. We venture to add, that, in these faulty qualities of style, it falls far short of many more medical publications than it surpasses. And we further add, that, in proportion to its extent, there is less superfluous verbiage in it, than in the tirade of the reviewer.

If there be, we say, in the "Elements," any amount of redundant matter—any thing we mean that does not belong to Pathological Anatomy, and that is not calculated to increase the value of a work on it, the reviewer is again invited to point it out. We urge him the more earnestly to this effect, in consideration in particular of the sentiments he has expressed on two occasions. Pathological Anatomy he observes, "takes cognizanse of *every morbid alteration going on in the economy.*" If this statement be correct (and we admit it to be so) it gives to the outline of the science a latitude, which cannot, we confidently assert, be *redundantly* crowded by the *whole* of the contents of Professor Gross's "Elements," much less sufficiently filled by the *half* of them—the compass within which T. S. asserts the work should have been restricted. Again he observes;

"Should another edition (of the Elements) be called for, we trust the author will take advantage of this to *prune it of its redundances.*" Let him, we repeat, specify those redundances, and *prove* them to *be* so; and we venture to pledge ourselves for their removal, in "another edition" of the work, which we cannot doubt will be shortly called for. We ask T. S. the more especially to point out the surperfluous matter, because we are fully convinced, that nobody else will be keen-sighted enough to detect it. Objects well suited to the microscopic eye of the wren, escape the lordly glance of the eagle.

The reviewer is also challenged to analyze, in a scholar-like manner, a paragraph or two, promiscuously taken from the same publication, and show wherein they posses a superabundance of words.

In doing this, T. S. will act the part of a critic, and be so far held faultless at least, if not praiseworthy, in his vocation; while the course of sweeping and indiscriminate condemnation which he has pursued, in the case before us, constitutes him a *reviler*, and attaches to him the ignominy of that odious office.

From a willingness moreover to share in the trouble and labour, as well as in the responsibility of the critical analysis thus challenged and demanded, we here present T. S. with an extract from the "Elements of Pathological Anatomy," on which his acumen and skill in his calling may be fairly tested. It is taken from Vol. II p. 245, the first place, at which the book fell open, when we took it up, and is not therefore a selected passage.

"The symptoms of carcinomatous disease of the stomach are never so urgent when the lesion is seated near the cardiac extremity, as when it involves the pylorus. In most cases, the signs are those of chronic irritation; that is to say, the patient is affected with indigestion, lancinating pain, constant acidity and flatulence, and occasional vomiting. There is considerable emaciation, with gradual wasting of the muscular powers; the eye is sunk, the complexion sallow, and, toward the last, the matter ejected from the stomach has a very offensive smell, and a dark colour like coffee-grounds. These symptoms it is obvious are not diagnostic; nor should they ever be so regarded, unless the schirrous tumor be at the same time perceptible by the hand placed upon the abdomen."

We repeat our request that T. S. will be so obliging as to enrich us, with a portion of the stores of his critical skill, by pointing out the redundancy of style in this paragraph—But he will not, because he cannot bestow on us the favour. The style is *not* redundant. Yet it is the common style of the work.

If then he cannot point out, in Professor Gross's work, a redundancy or irrelevancy of either matter or style, on what ground has he pronounced it *too voluminous, by half its size?* The answer is plain, and may be easily rendered. He is hostile to the work, or its distinguished author; or perhaps to both—or possibly to the school in which the author was educated—or to that in which he now holds a chair.—Or has he set a *price* on the "hebenon" of his inkstand, and penned his effusion for *pecuniary hire?*—If neither a part nor the whole of these motives, be the cause of the calumnious assault by T. S. on Professor Gross's Elements, we leave to himself to avow the cause, or to conceal it at option—wholly regardless of the course he may adopt.

Though we could easily cite, perhaps by the dozen, other impertinent and insulting passages, in the reviewer's pasquinade, we would hold ourselves unjustifiable in dwelling on them any longer. Nor should we have ever condescended to notice them at all, on account of any qualities inherent in themselves. All that is *intrinsic* to them is but matter for contempt; and, in a case like the present, that sentiment would be most becomingly expressed by silence. They are indebted, for our notice of them, exclusively to the Journal, whose pages they disgrace. Nor will even that Periodical, NATIONAL as it is proclaimed to be, by those who have the most *substantial* interest in it, be able long to give weight or influence to articles so rank in falsehood, and so encumbered by other repulsive qualities. Instead of its elevating them to the level it should strive to maintain, they will, if frequently admitted into its pages, inevitably drag it down to their *own level.* By dint of talents and learning alone, no Journal can rise to distinction, and maintain its standing. Unless it be founded in truth and justice, and conducted with courtesy, its fall is inevitable. Partiality in science is, if possible, even more reprehensible and repulsive, than denunciation in politics, or persecution in religion.

But we are not yet done with T. S. and his review. We have now to advert to a few of the mistakes and blunders in doctrine, opinion, and reference to authorities, which he has committed, in the course of his animadversions on Gross's Elements. And here, instead of treating him in a spirit of retaliation, by meting to *him*, as he has meted to *others*, we shall extend to him that measure of justice and fairness, which he has withheld from the author of the AMERICAN SYSTEM OF PATHOLOGICAL ANATOMY.

Our remarks under this head of our subject we shall preface by observing, in relation to the charges by T. S. against Gross's Elements which we are now about to notice, that, were they all true, so abundantly petty and meagre are they, that they would amount to but very little, on the score of censure or condemnation. Their relative standing would be but that of spots on the sun, contrasted with the bright and multiplied masses of merit, by which they are surrounded; and which T. S. has passed over in silent neglect—perhaps because they are too extensive for the field of vision of his *microscopic eye.* We shall briefly advert to them, in the order in which they are presented in the review.

The first topic to which we shall here refer, is one in which the reviewer charges Professor Gross with error, in his views respecting tuberculous matter. The charge embraces three or four several points—the ingredients or elements of which that matter consists—whether it is originally deposited in a liquid, or a solid form—what, on the first of these points, is the opinion of Gendrin—and whether Broussais considered tubercles to be "lymphatic ganglions" enlarged by inflammation. On each of these points the reviewer contends that Professor Gross has involved himself in error; although the Professor has given an express opinion on only *one* point out of the four; and in that *he is correct.* It relates to the opinion of Broussais respecting tubercles;—whether or not they were held by him to be glands preternaturally enlarged. And if T. S. will turn to that distinguished writer's History of Chronic Phlegmasia, Vol. I, p. 42, Hays & Griffith's translation, Philadelphia, 1831, he will there learn that the author *did* regard tubercles as nothing more than *enlarged lymphatic ganglions.* We beg him to reperuse the volume, for his farther instruction (if indeed he has ever perused it) and save us the trouble of extracting the passage.

As regards the opinion of Gendrin on the composition of tubercular matter (the leading object of the reviewer being, from his own acknowledgement, to fasten error on Professor Gross in relation to that point) the following is all that the "Elements" contain on the subject. And it is a mere *narra-*

*tive* or *statement of facts*, containing nothing in the form of *positive opinion*—nor even of hypothesis.

"The chemical, physical, and anatomical characters of this matter (tubercular) all conspire to show its similarity with the coagulating lymph, as it is revealed to us upon the free surface of the serous and mucous textures, in the various splanchnic cavities, upon the surface of a recent wound, or a granulating ulcer, and finally upon the surface of the crassamentum of blood, taken from blood affected with inflammation. From the well-conducted researches of Dowler, *Gendrin*, and Bretonneau, it clearly appears that the substances found in these different situations are all composed essentially of albumen, fibrin, and gelatin, in varying proportions, there being sometimes a predominance of one, sometimes of the other."

This, we repeat, is all that Professor Gross has said respecting Gendrin and his opinion, touching the composition of tubercular matter. And if T. S. can see in it aught that is unjust, or in any way exceptionable toward that distinguished pathologist, he is much more lynx-eyed than we are. Whether Gendrin subsequently changed his views on this subject, we do not now remember. Nor, immaterial as it is to the point at issue, shall we trouble ourselves to inquire.

As respects the contest on the form or condition, in which tubercular matter is originally deposited—whether liquid, semi-liquid, or solid—we shall only observe, that, if not frivolous, it is certainly *bootless*, in the present state of pathological science. The immediate mode of nutrition and growth, morbid as well as healthy, is as yet a concealed point, which neither T. S. nor ourselves are prepared to disclose. We shall therefore decline all effort toward that effect; and it will not perhaps be unwise in him to do the same—at least until farther research shall have better qualified him for the intricate task—or until he shall have duly resorted to animal magnetism, or some other new fangled source of revelation, and knowledge. His *prevoyance* on the subject at present, we have reason to believe is exceedingly limited. We shall only add, that nothing short of a *settled resolution* to make war on Gross's "Elements," should it be even a *musquito-war*, could have tempted T. S. to labour in a matter so puny and unprofitable.

The cutaneous affection, *porrigo*, is made the ground of another attack on Professor Gross and his "Elements." Respecting that disease the reviewer pronounces him mistaken on two points—the classification of it—and the opinion that it is contagious.

Here again nothing is positively settled by pathologists—some favouring and others opposing the classification of Professor Gross; and some pronouncing the affection contagious, while others deny it the possession of that quality. And, in professional eminence, and perhaps also in number, the contending parties are about equal. The Professor therefore only concurs with one party, and the reviewer with the other. His *own self-supposed infallibility* moreover excepted, the latter has adduced no shadow of proof that he is right, and the former wrong. And fortunately we live in an age and a country, in which the apothegms, that the *Pope is infallible*, and that the *King can do no wrong*, are rejected as assumptions, marked alike with inanity and arrogance. Nor is it probable that the pretensions of T. S., the reviewer and collaborator, to the same effect, will be received with more favour, or spurned with less contempt. When *he* shall have learnt to rely on fact instead of fancy, and to deal in demonstration instead of dogmatism, *others* will perhaps rely on *him*—not before.

On the diseased condition in general of the arachnoid membrane, respecting which Professor Gross is charged by the reviewer with sundry mistakes, we are not prepared to speak with confidence; because we have not satisfactorily studied the subject. As regards one point in the matter, however, we *are* thus prepared. Long before the researches of Drs. Gerhard and Rufz, all reading and enlightened physicians, were familliar with the fact of the frequent existence of tubercles in the arachnoid membrane, accompanying their existence in other tissues. Wherefore then has T. S. so boldly asserted, that to those two pathologists (Drs. Gerhard and Rufz) "we are especially indebted for our knowledge of *the coexistence of tubercles* of the archnoid with those in other parts of the body!" Was it because he knew no better, and supposed other people as ignorant as himself? was he himself more correctly informed, but fancying others to be ignorant, did he attempt to palm on them what he knew to be a falsehhod? did he write merely to eke out a sentence, reckless alike of its falsehood or truth? or did he act in obedience to some other motive more congenial to him, because more dishonorable? We await his reply, and shall believe or not, according to circumstances.

Of the merits of Dr. Gerhard, as an ardent and successful cultivator of pathological anatomy, none think more highly than we do, or are more willing to award to him the commendation he deserves. And the measure of that commendation we acknowledge to be ample. But we cannot consent to be witnesses of an unjustifiable project to use his name, in derogation of the well-earned reputation of Professor Gross, without coming promptly to the rescue, and earnestly endeavoring to avert the mischief. Nor has Dr. Gerhard, we feel confident, countenanced the project. In confirmation of our belief to this effect, we are gratified in being able to refer to a notice of the "Elements," very different in character from that by T. S., which appeared, some time ago, in the "Medical Examiner," and has been generally attributed to Dr. Gerhard's pen.

Another charge against Professor Gross, designed to be rendered as disparaging to him, as possible, is, that he has "*compiled loosely*" and incorrectly on the softening of the brain; because he has alledged that M. Rostan has ascribed it to *inflammatory irritation*. Now, in proof of the correctness of such an allegation, on the part of the Professor, we submit to the reader the following extract, from a Paris edition of Rostan's work.

"Malgré toutes les raisons que vous venons de donner en faveur de la natur inflammatoire du ramollissement cerebral, nous ne saurions croire qu'il soit *constamment* l' effet d' une inflammation."

What, we ask, is the true interpretation of this extract? and but one form of answer can be returned; which is to this effect. Though M. Rostan did not believe that softening of the brain was, in *every case*, the result of inflammation of that viscus; he admitted it to be so in *most cases*. If such be not the true exposition of the clause, we resign all pretension to an ability to expound.

Another topic on which the reviewer enters a grievous complaint against Professor Gross, is the *obliteration of the bronchiæ*. In a memoir on that form of disease, M. Reynaud attributes the obstruction of the tubes to pressure from without—the presence of foreign bodies within their cavities—and, in certain cases, to what he calls a "coarctation" of their walls, and a transformation of them into solid impervious cords. For this transformation Reynaud confesses that he cannot even *fancy* a cause. But possessing more decision of character, Professor Gross has the intrepidity to believe that he readily *finds* a cause in *inflammatory irritation.* To this he ascribes, with entire correctness, as we believe, the obstruction of the tubes, by the thickening of their mucous lining, and by fibrinous adhesion of their internal surfaces. This promptitude of Professor Gross T. S. calls, with a due amount of ancient wisdom and gravity, a *cutting of the Gordian knot.* The reason of his dissatisfaction is plain. He defers to no authority in medicine, except that of a *foreigner* or a *Philadelphian.* And, least of all, will he defer to a *man of the West,* whose authority he probably reads wrong end foremost, and considers it even an *evidence of error,* from his classical remembrance, that, among the ancient Romans, "*Punica fides*" meant deceit.

Seriously; to what else than inflammatory action, acute or chronic, can T. S. or any other medical casuist ascribe the obstruction of an air-tube from a thickening of its mucous lining, fibrinous adhesion of its internal surfaces to each other, or a contraction, or, if he likes the term better, as being more *outlandish,* a *coarctation* of its walls? We should be gratified by his reply; because it might suggest to us something new; as we confess ourselves at a loss for any cause other than inflammation, for the production of such effects. But perhaps he is afraid to reply to our question, lest, in so doing, he might subject himself to M. Rostan's rebuke, of which he tells us, on account of analogical reasoning. If so, his condition is pitiably barren in promise. For, to whatever extent his frigid cautiousness may protect him from error, it will never render him a successful discoverer of truth. The efficient explorer of the *terra incognita* of science, must be as fearless and enterprising as a pioneer of the wilderness.

In another part of his review, T. S. comes down on Professor Gross with an avalanche of wonder, and no less of censure, because he declines believing, in direct opposition to the evidence of his own senses, that a hepatized lung always and necessarily sinks in water. We say "in opposition to the evidence of his own senses;" and we mean what we say. On this subject, the Professor is not to be regarded, and spoken of, as a mere *reader*. He is also an *experimenter;* and we know that he has experimented *extensively*, and we have reason to believe accurately; because a turn for accuracy, if we mistake not, is one of his master qualities. And for the *truth* of his statements he is *above suspicion*. Under these circumstances, we cannot hesitate to admit the entire truth of the following extract, taken from a letter, received from the Professor within the last few days.

"To be serious, I can solemnly assure you, that, up to the publication of my work" (the Elements of Pathological Anatomy) "I never witnessed the sinking of the lungs in water, from hepatization, *in a single instance*, notwithstanding that I made repeated trials, both with the lungs of the human subject, and with those of the inferior animals, especially the horse. *Since that time* I have witnessed the circumstance *once*."

In this case Professor Gross has chosen to believe and report what he has himself done and witnessed, rather than what he has only read of, and heard of from others. And every man of independence and intelligence does the same—We have said that the Professor has "*chosen* to believe." But we retract the word "chosen," and replace it by *been compelled*. To man the clear testimony of his senses is *omnipotent*. His mind, if it be sound, can no more resist it, than his body can endure the thunder-stroke with impunity.

Much might be said, in exposition of this subject. But we may not dilate upon it. With the experiments of Professor Gross, some deceptive circumstances *might* have been connected. But it is equally probable that deceptiveness might have hung on the experiments of others. The question therefore of the sinking of hepatized lungs in water is still *on trial*, and can be settled only by subsequent experiments. And before T. S. assumes again consequential airs, or stalks into dogmatism on the subject, it might not perhaps be amiss in him to exchange the character of a rude and conceited reviewer, for that of a modest and patient inquirer.

In noticing the attack of the reviewer on Professor Gross, for some reputed mistake or blunder, on the subject of *interlobular* emphysema, we shall only remark, that the blunder *is his own*—whether *intentional* or *inadvertent*, we shall not pause to inquire. By misquoting the Professor, he has converted a clause of *good sense* into one of *nonsense*—and then made his comments on his *own handiwork!* Let him examine the passage, and he will find us correct. Such is his triumph, and such the means of its achievement! And they are both alike worthy of him.

The next attack on Professor Gross, by the reviewer, is on the subject of *pulmonary apoplexy*. And, may we judge of his meaning by his remarks, we can hardly persuade ourselves that he has any clear or definite knowledge of the real import of the terms he employs. It seems scarcely probable to us, we mean, that he understands correctly what it is that constitutes pulmonary apoplexy. If he does thus understand it, we certainly do not understand him.

In illustration of a position of which he is treating, the Professor describes one of the best defined and most striking cases of pulmonary apoplexy that stands on record. And the reviewer, in a manner peculiarly offensive, denies that it is pulmonary apoplexy at all. The ground however of his denial he consequentially conceals—intimating perhaps that his very thoughts, though he gives them no tongue, speak notwithstanding with marvellous eloquence, and convincing argument! In proof, as far as the highest authority can be so considered, that the disease recorded by Prof. Gross, was a case of genuine pulmonary apoplexy, Prof. Cruveilhier, of Paris, has described affections precisely similar, and bestowed on them the same appellation. The descriptions are contained in the Dictionaire De Medicine et De Chirurgie Pratique, published in 1829, and are as follows:

"It is not uncommon to meet in pulmonary phthisis, *especially in consequence of hemoptysis*, large apopletic accumulations, around tubercular masses, in different stages of their development." Again says Cruveilhier:

"There are *consecutive pulmonary apoplexies*, as, for example, when an aneurism of the aorta bursts into the substance of the lung. The blood which under these circumstances diffuses itself through the parenchymatous structure, is *either expectorated in very great quantities*, or it escapes into the pleuritic cavity."

Yet, in the very face of these, and many other similar statements that might be adduced, T. S., if we do not misunderstand him, denies that pulmonary apoplexy can be produced by a *hæmorrhagy*, especially if the blood flows from the rupture of a large vessel. As if cerebral apoplexy is not frequently the result of *extravasated* blood compressing the brain, as well as of blood preternaturally accumulated in unruptured vessels!

So preposterous, we repeat, is the notion we have here imputed to the reviewer, that we are positively apprehensive of having mistaken his meaning. If so, the mistake is *real*, not *affected*. For we disavow all intention of doing him injustice.

The reviewer, being apparently quite anxious to make a parade of his intimacy with every thing that bears relation to

M. Louis of Paris, has censured Professor Gross's work, on account of its misdating a memoir by that gentleman, on the softening of the gastroenteric mucous tissue. *And the mistake was but a trivial misprint,* which arose from the fact that the Professor did not himself correct the proof sheets of his "Elements," in their passage through the press—the work being printed in Boston, while he was in Cincinnati. For this the reviewer, resolved to collect and proclaim to the world every error and inaccuracy of the publication, whether great or small, accidental or otherwise, had not the liberality (we should rather say the *justice*) to make the slightest allowance. Hence his carping complaint, that the memoir of M. Louis, just referred to, and which was *really* published in 1825, is stated in the Elements to have been published in 1829! This, we repeat, is an *error of the press.* But, in the mean time, T. S. committed a *positive* error *of his own,* by asserting that the memoir of M. Louis was printed in 1826!—This, we acknowledge, is altogether an exceedingly *small* affair. And we have condescended to notice it only to expose the captious temper of the reviewer, and the grovelling kind of warfare which it was his pleasure to institute, against, the "Elements of Pathological Anatomy." We beg the reader to excuse us for the trouble we have given him in perusing the statement. Nor will he perhaps very seriously condemn T. S. on account of the Lilliputian size of his *war-weapons,* but make for him all due allowance, especially when he recollects that *little things are great to little men!*

A few remarks on one point more of cavil and censure by the reviewer, and we shall be done with the subject. It is his accusation of Professor Gross of having neglected to notice, with the fulness and respect to which they are entitled, the discoveries of Dr. Gerhard in Pathological Anatomy.

And his leading reason—that one at least which he urges most strenuously, why such notice ought to have been taken is, that *Dr. Gerhard is an American.* Nor do we either deny the soundnes of that reason, or wish, in any degree, to detract from its force. On the contrary, we admit it, in both respects, to its full extent.

In the present case, however, it is a two-edged sword, cutting in each direction, and falling, (to say the least of it) with fully as much force on the reviewer, as on Professor Gross. The latter gentleman is an American, as well as Dr. Gerhard. Why then did not T. S. remember this, during the time of the composition of his review, and allow the recollection to mitigate, in some degree, the spirit of detraction, with which he so unsparingly assailed the Professor! Does he regard it as a mark of disrespect or hostility in Professor Gross toward Dr. Gerhard, that he did not do *entire justice* to his pathological labours and discoveries? How loud would have been his complaint then, and how fierce his denunciation of the former gentleman, had he not only neglected the latter, and withheld encomium from him, but made him a subject of disparagement and calumny! In such a condition of things, may we judge from what has already occurred, the resentment of the reviewer would have been incontrollable and boundless, venting itself, if not in the most boisterous, at least in the deepest and deadliest anathemas. Of Professor Gross's failure then in respect and justice toward the Americanism of Dr. Gerhard, (if indeed he *has* failed) T. S. ought to be the last person in the community to utter a complaint. The Professor moreover is still further censured, because, while he passes Dr. Gerhard, though an American, with but little notice, he quotes respectfully European writers of less merit. To this we reply very confidently, that however courteous Professor Gross may be, in his references to foreigners, he defers to none in the style of *semi-adoration*, which the reviewer manifests especially toward *Dr. Louis*, whom he all but *idolizes!* In truth, though the standing of *that physician* is deservedly high, and his merit, *in some respects*, unsurpassed, the amount of incense burnt to him is excessive. And if we are not mistaken in the import of certain indications, which we think we perceive, his popularity and sway in the schools of Paris, are already on their wane. He is too *exclusive* in his doctrines; and, while professing to have *no theory*, he is one of the most inveterate and uncompromising theorists and dogmatists of the day—or of any day. And, as a *practitioner*, he has the reputation of being *proverbially unsuccessful.* Still is he, especially in the estimation of a certain American sect in medicine, the *beau ideal* of a finished æsculapian!

A few observations on *the particular discovery* of Dr. Gerhard, for his silence respecting which Professor Gross is most severely censured by the reviewer, may not be amiss. It is that of the *actual distinction* between *typhous* and *typhoid* fever.

Is it true, that Dr. Gerhard has made this discovery? Is such discovery, whether made by him, or by any other inquirer, *positively in existence?* Is the *essential* distinction between those two forms of fever *more certainly* known now, than it was at the beginning of the nineteenth century? Stronger still; *is* there an *essential* distinction between them? Or are they any thing more than different forms of the same complaint? That these questions, if propounded to different pathologists of the highest standing, would elicit different and contradictory replies, cannot be questioned. Already have sentiments been uttered by such pathologists, in all respects tantamount to such replies. The question therefore is yet unsettled.

Our favourable and high opinion of Dr. Gerhard has already been openly and sincerely expressed. And we here reiterate it. But we hold it at least exceedingly doubtful, whether his discovery, in the pathology of typhoid fever, of what is regarded as the *cause* or *root* or *essence* (or by whatever other name it may be called) of that complaint, be any thing but the detection of a *concomitant* of it—a mere lesion *produced by* the disease, instead of *itself producing* the disease.

That certain intestinal glands are found to be *generally* in a morbid condition, in patients who have died of typhoid fever, is not denied. But is it certain that they are *as generally* in such condition, at the commencement of the fever? and produce it through the instrumentality of that condition? No, it is not. On the contrary, there is reason to know that the glands referred to are *not, in every case*, morbidly affected at the beginning of the complaint; and that they cannot therefore, in every case, act as its cause. In corroboration of this, it may be mentioned, that, in some cases of typhoid fever, in which the most careful inspection was held after death, the glands of Peyer were in a natural condition—neither ulcerated, inflamed, nor in any perceptible degree deranged. And even *one* such case is *fatal to the doctrine*. The *cause must* be present, else the *effects* can never show themselves. And this is as true of disease, as of any thing else. The existence of a single case therefore, we repeat of true typhoid fever, without any morbid affection of the glands of Peyer, proves conclusively, that when such affection occurs, it is a concomitant or symptom of the disease, instead of its cause. Besides; it would be no easy matter even to *conjecture*, in what way the morbific agent, productive of typhoid fever, be it what it may, can reach Peyer's glands, without injuring other parts in its passage.

The existence moreover of a radical and essential distinction between typhoid and typhous fever, is by no means established. Far from it. Assuredly the external manifestations or symptoms of the two affections do not lead to a belief in such existence. They are much more favorable to the doctrine of their being but different forms and degrees of the same affection. Typhous and typhoid fevers exhibit fewer and less striking traits of dissimilarity, than do *quartan* and *quotidian* intermittents; or than scarlatina *simplex* and scarlatina *maligna*. Yet are the varieties of the *two latter* diseases considered as *only varieties* of two complaints, instead of being themselves *original complaints* essentially different.

Again; Dr. Gerhard's investigations of the pathology of typhoid fever have been limited, if we mistake not, to the Blockley Hospital, near Philadelphia. But that great and excellent establishment is an asylum of charity. Most of the patients therefore, that crowd its wards, are paupers, shattered, exhausted, and debased, by habits of intoxication, and indulgences in other sorts of intemperance and debauchery. That these agents therefore must act as modifying causes of their maladies cannot be doubted. Hence, though Dr. Gerhard may have unfolded, and no doubt has, *somewhat* of the pathology of the typhoid fever of Blockley Hospital; it by no means follows that he has disclosed the *entire pathology* of that complaint in every hospital and locality where it may prevail. It is not at all certain, we mean, that he has disclosed what we would call its *essential* pathology. For, that diversities of climate and country, situation and condition of life produce, in the same diseases, great diversities in symptoms and lesions, is a maxim as undeniable, as that which affirms things equal to one and the same thing to be equal to one another. As yet the pathology of typhoid fever is known *but in part.* And whatever benefits Dr. Gerhard's researches on that subject may have conferred on science, or whatever honors they may have won for himself, (and none of his countrymen are less inclined than ourselves to cast a shade on either) it may notwithstanding be found hereafter, that more importance has been ascribed to his *discoveries*, than they actually merit.

Once more. In still farther corroboration of the opinion just advanced by us, we are sustained by positive and high authority in asserting, that Peyer's glands have been found deeply deranged, in cases which had not been marked by a single symptom regared as *pathognomic of typhoid fever.*

Finally: we are aware of the existence of a strong probability, that, in consequence of the spirit and sentiments which mark this paper, we shall be charged, by certain characters, with the design of kindling the fire of discord between the medical Faculties of the eastern and western States, and arraying them against each other, as partisans at least, if not as actual and open enemies. But the charge, if made, will be utterly groundless. One of our leading objects is, to rebuke the principles, defeat the measures, and avert the meditated mischief of the Philadelphia *clique*, to which we have already adverted; and the effect of whose *conspiracy*, if not checked and extinguished, will inevitably be, to produce the discord and hostility, to which reference has been made.

Our earnest and fixed desire is for peace, harmony, and fraternal regard, between the physicians of the East and the West. And for the promotion of that most desirable state of feeling and relation we are determined to labour. But its basis must be formed of the proper materials, else it will be worse than warfare, and cannot endure. It must be founded on justice, mutual respect, and an equality of rights. There must be no affected superiority, or domineering spirit manifested in the East, under the expectation of meekness, submission and tolerance in the West. Reviewers *there*, must not, because they reside on the waters of the Atlantic, so far transgress the landmarks of their calling, as to convert *criticism* into *calumny*, in relation to medical productions *here*, merely because they have been written on the waters of the Mississippi.

In a word, let the great and respectable body of the medical Faculty in the eastern States, rebuke the insolence, and restrain the licentiousness of all such traductive reviewers as T. S., that may maliciously hiss and flicker their forky tongues among them, against western medicine—let the Faculty of the East do this, and none shall more promptly than ourselves extend to them cordially the hand of friendship—and never withdraw it again, except for a reason that shall justify the act. But, as long as their *so-called* reviewers shall be permitted by them, unadmonished and unchecked, malignantly and slanderously to assail medical writings and their authors in the West, we shall continue our aid to *chastise* them, and our endeavours to *crush* them. And, as far as their influence, on the waters of tho Mississippi is concerned, they *will* be crushed.

It was our intention to have noticed in this paper certain points of doctrine and opinion, which are found both in the "Elements" of Professor Gross, and the *tirade* of his assailant, in which we are not prepared to concur with either party. But we have already so far transgressed the bounds allotted to us, that we must here close our article, and shall probably resume the subject on a future occasion. C. C.

